# Editorial: Metabolomics perspectives for clinical medicine, volume II

**DOI:** 10.3389/fmolb.2025.1577050

**Published:** 2025-03-13

**Authors:** Danuta Dudzik, Julia Kuligowski, Víctor González-Ruiz, Hector Gallart-Ayala

**Affiliations:** ^1^ Department of Biopharmaceutics and Pharmacodynamics, Faculty of Pharmacy, Medical University of Gdańsk, Gdańsk, Poland; ^2^ Neonatal Research Group, Health Research Institute Hospital La Fe (IIS La Fe), Valencia, Spain; ^3^ Centro de Metabolómica y Bioanálisis (CEMBIO), Facultad de Farmacia, Universidad San Pablo-CEU, CEU Universities, Madrid, Spain; ^4^ Metabolomics and Lipidomics Facility, Faculty of Biology and Medicine, University of Lausanne, Lausanne, Switzerland

**Keywords:** clinical metabolomics, biomarker discovery, chronic diseases, personalised medicine, translational medicine

Metabolomics, including lipidomics, is a rapidly evolving field that profoundly impacts clinical medicine. It facilitates precise disease diagnosis, personalized treatment strategies, and the identification of novel biomarkers and mechanisms of pathogenesis. Advancements in mass spectrometry and computational approaches have enabled the detection and identification of metabolites and lipids in complex biological samples, providing a comprehensive biochemical profile of an individual’s health status. Unlike genomics, which reveals genetic predisposition, metabolomics captures the actual dynamic interplay of biochemical pathways, environmental influences, and disease progression, making it a powerful tool for clinical applications (Mohr et al., 2024; Singh et al., 2023; Misra, 2020; Marques et al., 2024). This Research Topic, “*Metabolomics Perspectives for Clinical Medicine: Volume II*,” brings together seven original research articles, one opinion, and one review, offering diverse insights into the evolving role of metabolomics in clinical research.

The primary objective of the published original reports was to improve diagnostic accuracy and patient stratification by integrating metabolomic data with traditional clinical approaches. The study by Chamoso-Sánchez et al. focuses on identifying metabolic subtypes (or metabotypes) in childhood obesity using a multiplatform metabolomics approach. The study recognizes that childhood obesity is a complex condition influenced by genetic and environmental factors. Therefore, the objective was to improve patient classification beyond traditional clinical and genetic assessments. The application of factor analysis and hierarchical clustering resulted in the identification of three distinct metabolic subtypes, despite traditional genetic and clinical markers failing to classify these patients effectively. The findings suggest that metabolomics can improve patient stratification and support personalized treatment strategies, offering a more precise approach to predicting disease risks and treatment responses. In the articles by Godzien et al. and Sieminska et al., the role of lipids in non-small cell lung cancer (NSCLC) is explored, focusing on their potential as biomarkers for differentiating between adenocarcinoma (ADC) and squamous cell carcinoma (SCC) subtypes, and investigating the role of oxidized phosphatidylcholines (oxPCs) in NSCLC patients. In the study by Godzien et al., the authors observe that long-chain oxPCs (LCh-oxPCs) were the predominant form in plasma, whereas short-chain oxPCs (SCh-oxPCs) constitute the main fraction in tissue in NSCLC patients. The study highlights that oxidized lipids play a dual role in lung cancer, acting as both protective and harmful agents depending on their structure and concentration. LCh-oxPCs have been associated with protective functions in lung endothelium, whereas SCh-oxPCs contribute to cellular damage and inflammation, potentially driving tumor progression. The authors conclude that the SCh-oxPCs accumulated in the cancer tissue of NSCLC patients, due to their high toxicity, could be considered a potential therapeutic target. Sieminska et al. reveal differences in the profile of oxPCs and monoacylglycerol phosphatidic acids (LPAs) between ADC and SCC subtypes. The study by Lokhov et al. aimed to explore the application of a clinical blood metabogram (CBM) in early-stage Parkinson’s disease (PD) diagnosis. The primary objective was to assess the CBM’s ability to detect metabolic alterations in the blood characteristic of PD, providing a non-invasive diagnostic tool that could improve early detection and disease monitoring. The study employed direct-infusion mass spectrometry and principal component analysis together with metabolite set enrichment analysis to analyze blood samples. The findings indicate that CBM can effectively distinguish PD patients from healthy controls, with a diagnostic accuracy of 77%, a specificity of 71%, and a sensitivity of 82%. The authors conclude that CBM could be integrated into clinical practice for PD diagnosis, disease progression monitoring, and treatment evaluation, offering a promising avenue for personalized medicine. The novelty in the study by Dudzik et al. lies in the application of metabolomics to distinguish between early-onset and late-onset gestational diabetes mellitus (GDM) using both targeted and untargeted approaches. The study’s findings indicate that specific lipid and carbohydrate metabolism alterations are strongly associated with GDM, with distinct metabolic signatures differentiating early-onset and late-onset cases. These findings suggest that metabolomics can enhance GDM diagnostics by providing deeper insights beyond traditional glucose measurements and clinical markers, paving the way for more personalized treatment strategies. The research by Warmuzińska et al. investigates lipidomic alterations in kidney grafts during warm ischemia and preservation, utilizing solid-phase microextraction combined with liquid chromatography-high-resolution mass spectrometry. The primary objective was to evaluate how different preservation methods, including normothermic and hypothermic perfusion, affect lipid metabolism in kidney grafts. By employing a minimally invasive chemical biopsy technique, the study enabled repeated sampling of the same tissue, allowing real-time monitoring of metabolic alterations during the transplantation process. The study’s findings indicate that normothermic *ex vivo* kidney perfusion has a beneficial effect on graft function and the chemical biopsy technique allows tracking alterations in the graft throughout the entire transplantation procedure. Pietrowska et al., using targeted metabolomics based on the AbsoluteIDQ® p180 kit, investigate differences in concentrations of various metabolites in aqueous humor collected from both eyes of the same patients. The authors conclude that with a few exceptions, a single eye was representative of the fellow eye in terms of the concentration of most of the analyzed metabolites. The review by Ye et al. focuses on the role of glutamine metabolic reprogramming in hepatocellular carcinoma (HCC), a highly lethal liver cancer. The study highlights how altered glutamine metabolism supports tumor growth, immune evasion, and therapy resistance. The authors discuss how cancer cells rewire glutamine metabolism to fuel biosynthetic pathways, maintain redox balance, and activate key signalling pathways like mTORC1, which promotes tumor progression. Additionally, the review explores glutamine-related metabolites as potential biomarkers for early HCC detection and treatment response monitoring. The authors evaluate metabolic targeting therapies, such as glutaminase inhibitors, which aim to disrupt tumor-specific glutamine dependencies, offering new therapeutic opportunities. Finally, the opinion paper by Yang et al. discusses recent advancements in mass spectrometry imaging (MSI) for metabolomics and its potential impact on biomedical research and clinical applications. This powerful analytical technique enables the spatial localization of metabolites within biological tissues, providing valuable insights into disease mechanisms and metabolic alterations at the cellular level. Therefore, the authors highlight how MSI overcomes the limitations of traditional metabolomics by preserving spatial context, which is crucial for understanding tissue-specific metabolic variations in diseases like cancer and neurodegenerative disorders. Additionally, the paper explores the integration of MSI with other omics approaches, to achieve a more comprehensive molecular profile.

All studies highlight the potential of metabolomics and lipidomics in advancing personalized medicine by improving disease classification, early diagnosis, and targeted treatment strategies. However, all the authors recognize the challenges that remain in translating these findings into clinical practice, including the need for standardized methodologies, addressing environmental and lifestyle influences on metabolic phenotypes, and the need to improve data interpretation. Consequently, future studies should prioritize the validation of metabolic biomarkers, the optimization of analytical workflows, and the identification of patient subgroups that would most benefit from metabolic-based interventions. This, in turn, should pave the way for more effective and individualized healthcare solutions.
